# Clicking viruses—with chemistry toward mechanisms in infection

**DOI:** 10.1128/jvi.00471-25

**Published:** 2025-05-14

**Authors:** Urs F. Greber

**Affiliations:** 1 Department of Molecular Life Sciences, University of Zurich 27217, Zurich, Switzerland; Universiteit Gent, Merelbeke, Belgium

**Keywords:** click chemistry, DNA, RNA, protein, lipid, human immunodeficiency virus (HIV), herpesvirus, adenovirus, virus entry, virus genome packaging and assembly

## Abstract

Viruses subvert cells and evade host defense. They emerge unpredictably and threaten humans and livestock through their genetic and phenotypic diversity. Despite more than 100 years since the discovery of viruses, the molecular underpinnings of virus infections are incompletely understood. The introduction of new methodologies into the field, such as that of click chemistry some 10 years ago, keeps uncovering new facets of viruses. Click chemistry uses bio-orthogonal reactions on chemical probes and couples nucleic acids, proteins, and lipids with tractable labels, such as fluorophores for single-cell and single-molecule imaging, or biotin for biochemical profiling of infections. Its applications in single cells often achieve single-molecule resolution and provide important insights into the widely known phenomenon of cell-to-cell infection variability. This review describes click chemistry advances to unravel infection mechanisms of a select set of enveloped and nonenveloped DNA and RNA viruses, including adenovirus, herpesvirus, and human immunodeficiency virus. It highlights recent click chemistry breakthroughs with viral DNA, viral RNA, protein, as well as host-derived lipid functions in both live and chemically fixed cells. It discusses new insights on specific processes including virus entry, uncoating, transcription, replication, packaging, and assembly and provides a perspective for click chemistry to explore viral cell biology, infection variability, and genome organization in the particle.

## INTRODUCTION

Infectious diseases caused by viruses are ancient (1), yet viruses have only been known for some 130 years (2). Viruses have been a burden on individuals and societies and are difficult to control, as they are manyfold, occur in large numbers, and evolve in a short time (3). They are closely interlinked with their hosts (4–6). Viruses evade pressure from chemicals and host defense mechanisms, including innate and adaptive immune reactions. Viral evasion occurs by mutations in genes encoding viral protein-protein interfaces, as exemplified by the neutral pH-adapted common cold virus, rhinovirus (7), by mutating viral replicase, protease, or integrase in the case of human immunodeficiency virus (HIV) (8–10), or by altering viral surface epitopes to evade neutralizing antibodies (11). Genetic and phenotypic virus adaptations give rise to variable outcomes of infection, as manifested, for example, in a range of plaque shapes under laboratory conditions (12–14). Likewise, clinical phenotypes range from lytic infection and viremia (the systemic spread of virus across bodily fluids) to persistent infection with low virus spread, and abortive infections without formation of progeny particles and without viral dissemination.

A conceptual deficit in virology has been the poor understanding of cell-to-cell infection variability. Infection variability has been known since the 1940s (15), initially through pioneering studies of plaque assay with western equine encephalomyelitis virus and monolayers of chicken embryo fibroblasts under gellifying agarose (16), and more recently through fluorescence microscopy in aqueous medium (14). Besides big and small plaque phenotypes from genetically distinct virus variants (see for example, references 17, 18), clones of transformed human lung epithelial cells were recently shown to exhibit high or low susceptibility to adenovirus (AdV) infection owing to preexisting cell states with built-in memory rather than stochastic noise controlling the expression of the immediate early viral E1A gene (19). Considering that a cell state comprises a collective of biochemical, morphological, and contextual functions (20, 21), this challenge is only beginning to be addressed (22, 23). Resolving the impact of cell state on infection, single-cell analyses are required to reveal the heterogeneity of viral transcription, replication, and translation, for example, as shown with picornavirus RNA imaging and protein labeling (24), or single-cell RNA sequencing (scRNA-seq) for influenza virus (25) and HIV (26). For a comprehensive review, see reference 27.

The review here addresses how click chemistry can be employed for biochemical populations studies as well as single-cell analyses with single-molecule resolution, and sheds light into infection variability at the molecular level. A select set of viruses, including AdV, herpes simplex virus type 1 (HSV-1), and HIV-1, highlights how click chemistry informs on virus entry, assembly and egress, and DNA and RNA replication and transcription, as well as protein translation and lipid flux in infections.

## CLICK CHEMISTRY

The Nobel Prize in Chemistry 2022 to Barry Sharpless, Morten Meldal, and Carolyn Bertozzi celebrated click chemistry (28). Sharpless formalized the concept of click chemistry. Meldal and Sharpless, independently, put forward copper-azide-alkyne cycloaddition (CuAAC), an elegant and highly versatile click reaction in pure and applied chemistry enabling the development of biological probes, predominantly nucleic acids, and pharmaceutics (29, 30), while Bertozzi addressed the application of click chemistry for tracking glycans in living organisms (31).

**Fig 1:**
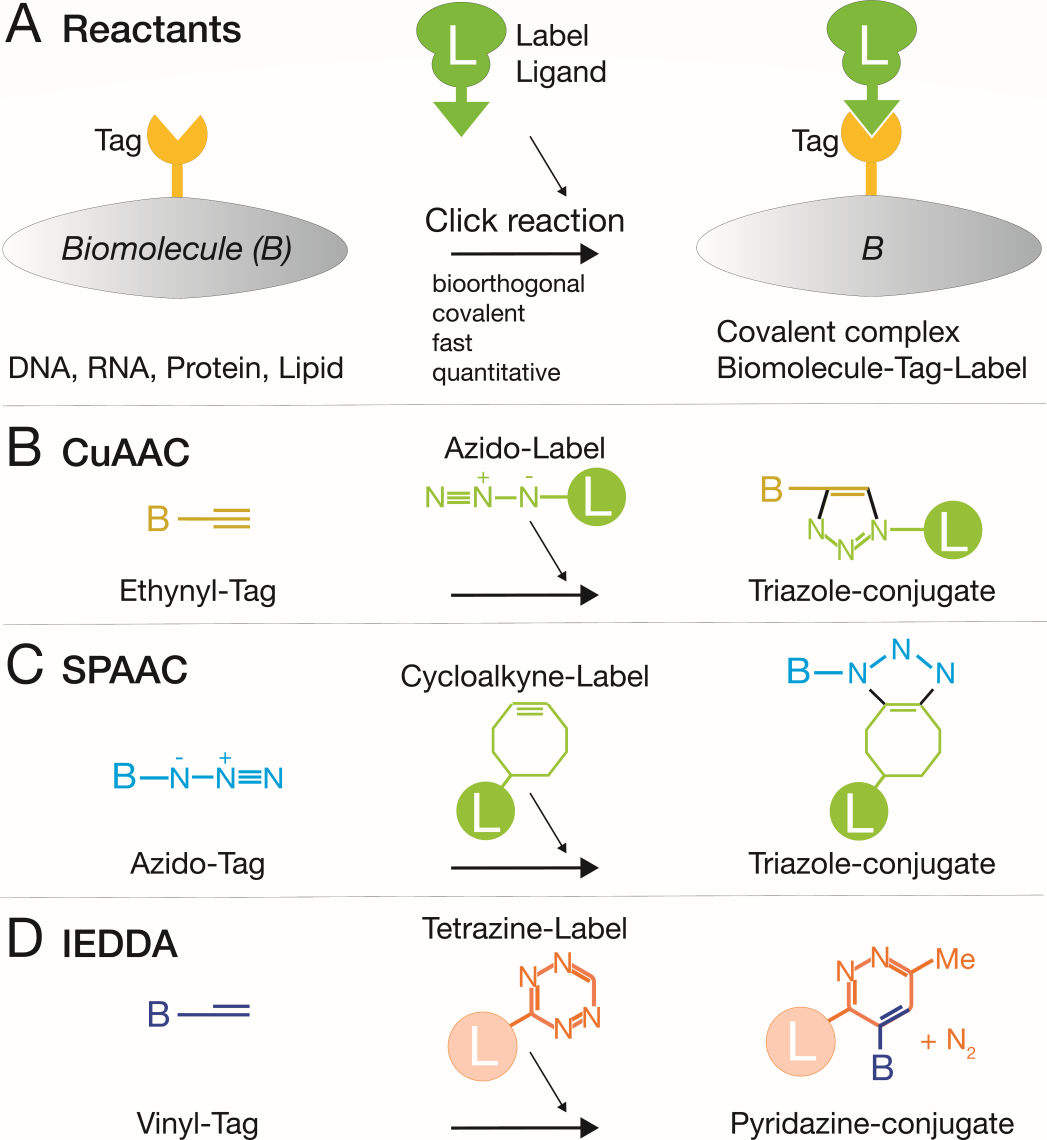
Overview of the most common click chemistry reactions in virus research. (**A**) Principal reactants. Chemically modified biomolecules (B), such as DNA, RNA, proteins, or lipids, contain chemical tags, such as alkynes, that react *in situ* with bio-orthogonally modified ligands, for example, azido-fluorophores or -biotin. This reaction yields covalent products containing tractable labels. (**B**) CuAAC couples ethynyl-tagged biomolecules to azido-labels yielding stable triazole conjugates. Suitable for chemically fixed cells. (**C**) SPAAC couples azido-tags to strained alkynes in live cells, yielding stable triazole conjugates. (**D**) IEDDA used, for example, to couple vinyl-tagged biomolecules to tetrazine-containing labels yields stable pyridazine conjugates suitable for live-cell studies.

Click chemistry engages rapid reactions and avoids unwanted side products. Its reactions are bio-orthogonal, that is, they are absent in unmodified cells. Principal reactants of click reactions with viruses are depicted in Fig. 1A. Click reactions occur under near physiological conditions and are not part of the normal repertoire of cells and viruses, and minimally interfere with cellular processes and infections (32–34). Their fast kinetics give rise to specific reaction products at high efficiency, low concentrations of reactants, and low toxicity (35). Different types of click reactions are used today; the most widely used one is CuAAC (Fig. 1B), although it is limited to fixed or lysed samples, because copper is intrinsically toxic to live cells (36). In living systems, it has been replaced by strain-promoted azide-alkyne cycloaddition (SPAAC, Fig. 1C) and inverse electron demand Diels–Alder reaction (IEDDA, Fig. 1D) (37–41). Click reactions covalently modify DNA, RNA, proteins, and lipids with a range of moieties, including fluorophores and biotin. Regarding click reactions used in the synthesis of anti-viral compounds, such as HIV protease inhibitors, the reader is referred to excellent reviews elsewhere (42–44).

## CLICKING VIRAL NUCLEIC ACIDS

The activity of viral nucleic acids, especially DNA and RNA genomes, determines if an infection is abortive, latent, persistent, or lytic. Monitoring viral nucleic acids is thus key to understanding virus infection variability, a major unresolved question in virology. To address infection variability, researchers have used labeled viruses, scRNA-seq and engineered cell lines, scRNA fluorescence in situ hybridization (scRNA-FISH), high-throughput fluorescence *in situ* DNA hybridization, or single-cell mass cytometry (45, 46). While RNA- and DNA-FISH provide single-molecule resolution (47, 48), the sensitivity of DNA-FISH for double-stranded viral DNA (vDNA) is limited (48, 49). Reasons comprise the harsh conditions required for DNA strand separation and the ensuing loss of signal, as shown by direct comparison with minimally invasive click chemistry using CuAAC in the absence of denaturation (32, 50). This exemplary case illustrates the power of click chemistry to unravel infection heterogeneity.

**Fig 2:**
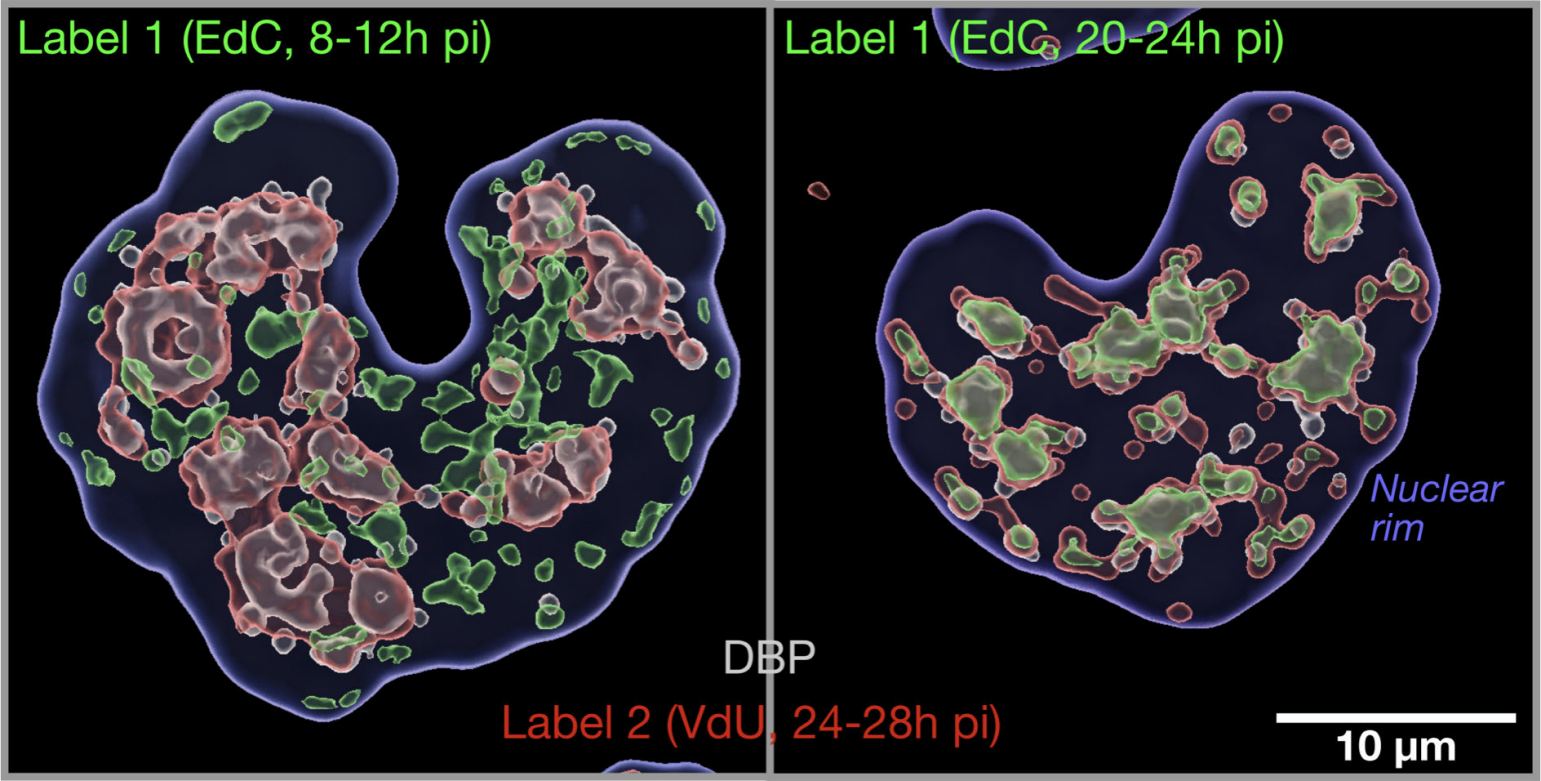
Imaging of vDNA replication dynamics by combined CuAAC and IEDDA pulse-chase click chemistry. Pulse-chase protocol with two bio-orthogonal click reactions, CuAAC and IEDDA. The former coupled ethynyl-modified deoxy-cytidine (EdC) to an azido-fluorophore, and the latter vinyl-modified deoxy-uridine (VdU) to a tetrazine fluorophore. The protocol allows for *in situ* distinction of vDNA synthesized early in AdV infection (early-replicated vDNA) compared to late-synthesized vDNA (late-replicated vDNA). The latter defines the viral replication center as indicated by immunofluorescence staining of the vDNA-binding protein (DBP, 72K). Note that labelling reagent 1 (EdC, green) was added to infected A549 cells 8–12 or 20–24 hpi, and label 2 (VdU, red) 24–28 hpi, followed by specimen fixation, DBP staining (gray), clicking with azide-AlexaFluor 647 (EdC) and AO-6MT (VdU), imaging in a Leica SP8 FALCON CLSM, and 3D rendering using Imaris10 (Oxford Instruments, Oxon, UK). The position of the nuclear rim was defined by staining with 4´,6-diamidino-2-phenylindole (blue). The images show the rendered volumes of infected A549 cell nuclei. Detailed procedures are described in reference 59. The images are courtesy of Dr. Alfonso Gomez-Gonzalez.

**Fig 3:**
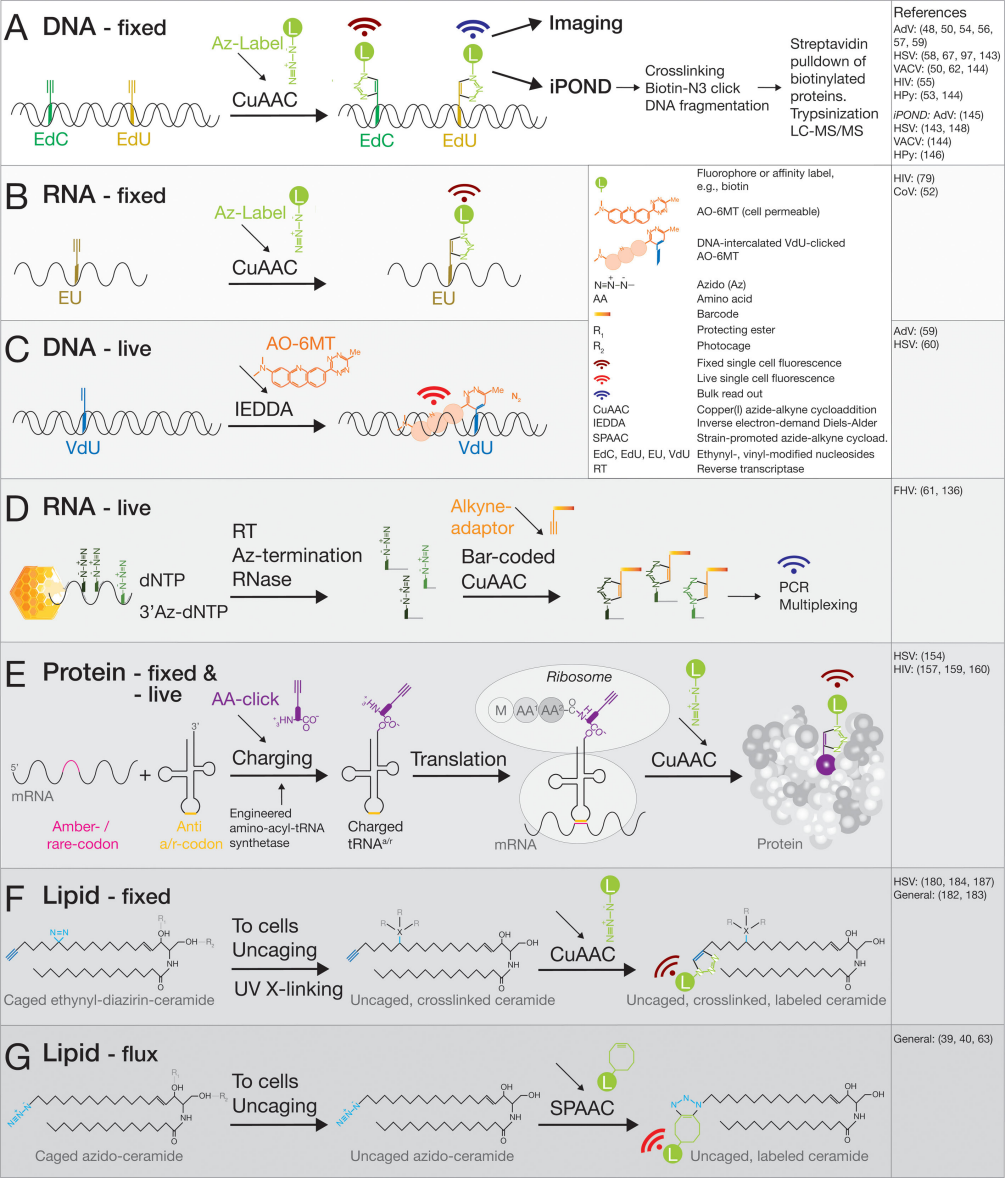
Schematic representation of a select set of click chemistry reactions with vDNA, vRNA, proteins, and lipids. (**A**) CuAAC-based detection of alkyne-tagged deoxynucleotides EdC or EdU incorporated into vDNA during viral replication. Azido-fluorophores or azido-biotin label the vDNA upon chemical fixation for analyses by fluorescence microscopy and iPOND (isolating proteins on nascent DNA) with mass spectrometry, respectively. (**B**) CuAAC-based detection of the alkyne-tagged nucleotide EU incorporated into vRNA, allowing for fluorescence microscopy analyses of newly synthesized vRNA. (**C**) IEDDA-based labeling of alkene-tagged deoxynucleotides (in this case, VdU) incorporated into vDNA, visualized with the DNA intercalating dye AO-6MT, which covalently links to the vinyl group by inverse Diels-Alder ligation. This allows for live-cell tracking of newly synthesized vDNA over several hours until the onset of cell toxicity. The labeling protocol developed for AdV is available in reference 59. An alternative protocol using IEDDA and strained *trans*-cyclooctene was developed for HSV-1 (60). (**D**) Population assay measuring stepwise heat-triggered vRNA exposure from flock house virus (FHV) particles. Exposed vRNA is random-primed with oligo-deoxynucleotides, reverse transcribed in the presence of chain-terminating 3’azido-tagged deoxynucleotides, which are stochastically incorporated by reverse transcriptase in the reaction mix, digested with RNase, tagged with an alkyne-containing barcode adaptor, and analyzed by ClickSeq and multiplexed PCR. This procedure mapped vRNA uncoating intermediates of FHV (61). (**E**) A minimal approach to click-tag proteins using genetically engineered bio-orthogonal tRNA and aminoacyl-tRNA synthetase to incorporate a noncanonical amino acid (ncAA) at a position of interest using an amber stop codon or a rare codon of choice. An alkyne group in the ncAA is covalently linked by CuAAC to an azido-fluorophore, or another reactive group, such as a tetrazine reacting with a strained functional group on the ncAA by IEDDA. (F, G) Multifunctional ceramide lipid probes for fixed as well as live-cell analyses of lipid localization and flux. Ultraviolet light irradiation of the diazirine group leads to photo-crosslinking by carbene insertion into nearby hydrogen-bonded carbon atoms. Esterified hydroxy groups masking their hydrophilic functionality enhance cell delivery of the probe and are removed by cytosolic esterases. Photo-destructible cages, such as coumarins or nitrobenzyl groups, prevent probe metabolization and can be released by blue light. The terminal alkyne tags on aliphatic chains can be coupled to azido- or strained-ligands by CuAAC and SPAAC reactions, respectively (62, 63).

The detection of vDNA and viral RNA (vRNA) in cells has traditionally been limited to just a few click reactions, including CuAAC and IEDDA (51). The combination of both CuAAC and IEDDA reactions in the same cell has recently been achieved, as depicted by a fluorescence micrograph of AdV infections (Fig. 2). This work was preceded by earlier studies that had incorporated 5-ethynyl-uridine (EU) into newly transcribed coronavirus RNA (52), 5-ethynyl-2´-deoxy-uridine (EdU) into human papillomavirus type 16 DNA (53), vaccinia virus (50), AdV (50, 54), or reverse-transcribed HIV DNA (55), while 5-ethynyl-2´-deoxy-cytosine (EdC) was incorporated into AdV and HSV-1 DNA (50, 56–59) (Fig. 3). CuAAC coupled the ethynyl-modified nucleotides in the viral nucleic acids to azido-fluorophores and revealed the vDNA and vRNA upon chemical fixation of the specimen (Fig. 3A and B).

One drawback of click chemistry in cells is that biosynthetic tagging of biomolecules often lacks specificity. For example, both viral and cellular DNA incorporate ethynyl-tagged deoxy-nucleosides, unless the nucleoside is added to cells when only cellular or vDNA is synthesized. The latter can, for example, occur in the case of AdV, HSV-1, or vaccinia virus, which shut off host DNA synthesis a few hours after infection (64–67). In the following sections, examples are discussed where specifically vDNA or vRNA was unambiguously tracked by single-cell imaging and bulk biochemical approaches using differential enrichment procedures to distinguish signals from cellular and viral DNA.

### Imaging

The nucleus compartmentalizes DNA and constitutes a hub for transcription, replication, DNA editing, repair, as well as chromatin assembly, condensation, and decondensation vital for cell function and survival (68). For example, *in situ* imaging has provided crucial information on transcription initiation, elongation, and processing (69), activation of origins in DNA replication (70), chemical modifications of DNA and associated histones in epigenetic activation and silencing (71), DNA repair (72), chromatin dynamics, or nuclear envelope breakdown and reformation in mitosis (73). Alone, the nucleus is also a hot spot for pathogen subversion, in particular DNA tumor viruses, retroviruses, or negative-strand RNA viruses replicating in the nucleus (74, 75). While HIV integrates a proviral reverse-transcribed vDNA into host chromatin, herpesviruses and AdVs give rise to vDNA factories in the nucleus, so-called viral replication compartments (VRCs) (76, 77). Below, we show how click chemistry has provided conceptual insights into these viruses.

#### Clicking HIV RNA and reverse transcription

A long-held assumption had been that reverse transcription of incoming diploid HIV plus-sense, single-strand vRNA occurs in the cytoplasm after uncoating from the capsid, and that multiple viral proteins including matrix, capsid (CA), nucleocapsid, integrase, and Vpr are part of the reverse transcription complex (78). Yet, the cone-shaped capsid also harbors reverse transcriptase. In incoming virions, vRNA tagged with EU became accessible to labeling depending on an early capsid uncoating step in the cytoplasm, in this case, onset of reverse transcription (79). Quantitative microscopy in cells incubated with deoxy-ethynyl-tagged nucleoside during HIV entry revealed EdU incorporation into reverse-transcribed HIV DNA and CuAAC conjugation of vDNA with a fluorophore, demonstrating that reverse transcription occurs within the capsid (55). Crystallography of CA hexamers, along with molecular modeling then provided support for this scenario by showing that each HIV capsid hexamer has a size-selective pore with a ring of six arginine residues (80). The arginine residues are crucial for high-affinity nucleotide binding, reverse transcription, and infection. In the absence of nucleotides, the pores are closed, suggesting that the pores are a dynamic gateway for nucleotides fueling capsid-based DNA synthesis. Such reverse transcription-active cone-shaped HIV-1 capsids were recently shown to be transported through nuclear pores by direct interactions with barrier-forming nucleoporins (81–84). Ensuing completion of reverse transcription and uncoating in the nucleus (85) is then followed by integration of the proviral DNA into host chromatin, preferentially to sites located near the nuclear envelope (86). In sum, click chemistry of incoming HIV RNA and reverse-transcribed proviral DNA has reinforced a paradigm change of reverse transcription from an ill-defined, laid open to a capsid-confined and well-shielded process that potentially affects virus uncoating, proviral DNA integration, and activation.

#### HSV-1 DNA tracking by CuAAC

Akin to HIV, herpesvirus DNA persists long term in infected cells with periods of silencing and reactivation. Infections with HSV-1 can be asymptomatic, mild, or life-threatening, and they last for the lifetime of the individual due to viral latency in neurons and sporadic dissemination upon reactivation (87).

Although members of all herpesvirus subfamilies have been tagged with ethynyl nucleosides and visualized by click chemistry (88), most click studies of herpesvirus DNA were reported with alpha-herpesviruses. For example, using CuAAC with ethynyl-nucleotides and azide fluorophores, glimpses into the decondensation of incoming HSV-1 genomes could be reported (89). vDNA was found to be imported into the nucleus independent of inhibitors of transcription, nuclear export, or proteasome, unlike other viruses, such as AdV, which requires nuclear export for docking to the nuclear pore complex (NPC) (90), and proteasome function for uncoating (57). Uncoated HSV genomes were found both in the nucleus and the cytoplasm (89), the latter likely representing misdelivered vDNA (91). The uncoated HSV-1 DNA had a larger volume than the packaged one (89). Likewise, the one in the nucleus increasingly decondensed with progressing infection, impeded by transcription inhibitors. At later stages of infection, HSV-1 genomes were located in VRCs, and there, they apparently decondensed to a larger degree than the incoming ones, notably with large genome-to-genome variability, possibly reflecting asynchronous biochemical processes on individual genomes.

Using CuAAC, the spatial organization of HSV-1 DNA synthesis was studied in multiround infections using a fluorescence tagging approach (67). These experiments revealed that uninfected cells around an initially infected cell induce the synthesis of their host DNA. Upon HSV-1 infection, these S-phase cells shut off host DNA replication and turned on vDNA synthesis. Although it was not assessed if the surrounding cells were more or less susceptible to HSV-1 infection, such a scenario would be in line with results from AdV infections, where cells inoculated in the G1 cell cycle phase (preceding S-phase) gave rise to higher immediate early viral transcription than G2/S/M phase cells (48). Collectively, the findings with HSV-1 resonate with an emerging concept where paracrine effector molecules not only lead to well-known autocrine and paracrine anti-viral states in case of interferon (92, 93), but might also have proviral effects in surrounding cells, as suggested in recent reports with human cytomegalovirus and severe acute respiratory syndrome coronavirus 2 (94, 95).

CuAAC was instrumental for identifying the interferon (IFN)-stimulated gene MxB restricting herpesvirus infections. Single-cell, single-genome fluorescence microscopy showed that EdC-tagged HSV-1 genomes are blocked from nuclear entry in MxB-expressing cultured human cells (58). The presence of capsids in the cytoplasm and the absence of capsid-free vDNA in the cytoplasm of MxB-expressing cells judged by fluorescence microscopy gave rise to the interpretation that MxB prevents the uncoating of HSV-1 capsids. Along the same lines, transmission electron microscopy showed that MxB-expressing cells had fewer empty capsids (lacking vDNA) and more electron-dense capsids (containing vDNA), without affecting the capsid numbers compared to MxB-nonexpressing cells.

An alternative interpretation was drawn from *in vitro* incubation of isolated HSV-1 capsids with cytosolic extracts containing MxB, suggesting that MxB disrupts HSV-1 capsids (96). Likewise, a study in HeLa cells suggested that MxB contributes to dismantling HSV-1 capsids and exposes EdC-tagged vDNA in the cytoplasm (97). Of note, the cytoplasmic click puncta were, however, difficult to detect next to prominent unspecific signals from the nucleus. This raises the possibility that capsid-free cytosolic vDNA was present at very low levels or subject to degradation by nucleases, while capsid-enclosed vDNA resisted clicking by CuAAC, as initially shown with isolated intact EdC-tagged AdV (50). This situation emphasizes the importance of alternative assays, such as PCR, to account for overall abundance of vDNA. Notably, PCR analyses of incoming EdC-tagged AdV showed a reduction of vDNA by about 30% between 3 and 7 hours post-infection, that is, before the onset of vDNA replication (48). This validated the nature of the DNA click puncta in the nucleus and cytoplasm as AdV DNA. Irrespective of the precise MxB mode of action against HSV-1, click chemistry showed that MxB expression prevents the nuclear import of incoming HSV-1 DNA and thereby protects uninfected cells from HSV-1 infection.

#### Live-cell clicking of HSV-1 by IEDDA

Herpesviruses are enveloped, double-stranded DNA viruses and replicate their large genome in the VRC of the nucleus (for reviews, see references 76, 98, 99). VRCs function through a network of proteins with intrinsically disordered regions mediating enrichment of select sets of viral and cellular factors for viral replication and transcription. How they are linked to vDNA encapsidation is an important open question. The dynamics of HSV-1 DNA replication were recently addressed by IEDDA using a triphosphate-pronucleotide (TrPP-Pro) linked to 2-*trans*-cyclooctene (2-TCO) and a lipophilic cleavable moiety for cell delivery (60). The 2-TCO-linked triphosphate analog of deoxy-cytidine helped to overcome the well-known limited substrate tolerance of host nucleotide kinases. TrPP-Pro-dCTP-2-TCO was incorporated into vDNA, and the highly reactive strained-alkene 2-TCO moiety could be coupled to a cell-permeable fluorescent tetrazine-tagged coumarin derivative, allowing for live-cell imaging of HSV-1 DNA, albeit at high background signal, so far precluding single-molecule detection.

#### AdV DNA in live cells

AdV delivers double-stranded DNA into the cell nucleus upon receptor-mediated endocytosis, endosomal escape, and nuclear transport of a capsid containing vDNA but lacking minor stabilizing proteins (100). The vDNA is uncoated from the capsid at the nuclear pore complex and imported into the nucleus, although a fraction of it is misdelivered to the cytosol (101–103), where it triggers the innate DNA sensor cGAS (104, 105). In the nucleus, viral early transcription activation goes along with remodeling of the viral chromatin, along with S-phase progression of the cell and inhibition of host DNA synthesis (106, 107). Consistently, the AdV DNA is remodeled by deposition of histone H3.3, a histone variant added to DNA independently of replication (108, 109). AdV DNA persists in the nuclei of immune cells where it expresses low amounts of the immediate early E1A protein, a key activator of the major AdV promoters (110–112). Upon derepression of early viral gene expression, for example, by immunosuppressive treatment or an ectopic viral infection, high levels of AdV gene expression, replication, and virion production give rise to life-threatening viremia (113, 114).

Live-cell detection of replicating AdV DNA was achieved by substituting the bulky trans-cyclo-octane (used with HSV-1) with the slim vinyl-tag in 5-vinyl-2’-deoxy-uridine (VdU) (59) (Fig. 3C). VdU was incorporated into the VRC, which comprises three viral proteins besides cellular factors, viral DNA-polymerase (DNA-Pol), single-strand DNA-binding protein (DBP), and terminal protein (77, 115). DBP is intrinsically disordered (116) and a key organizer of the VRC (117). VdU had previously been used for visualizing cellular DNA in fixed specimens through catalyst-free IEDDA alkene-tetrazine ligation (118). It had been adapted to live-cell imaging using high-affinity intercalation of a fluorescent tetrazine probe into VdU-DNA or 5-vinyl-uridine (VU)-RNA (119). Importantly, this strategy provided distinct DNA fluorescence above background through enhanced kinetic and fluorogenic features of acridine orange-6-methyl-tetrazine (AO-6MT). This dye dequenches upon intercalation with DNA or RNA. Remarkably, AdV DNA-polymerase readily incorporated VdU into the VRC and vDNA, with clear preference of VdU over 5-vinyl-2´-deoxycytidine (VdC) or 5-vinyl-2´-deoxyadenosine (VdA) (59). Whether vinyl-nucleosides incorporate into replication compartments of other DNA or RNA viruses can be explored.

VdU-AO-6MT-labeled AdV DNA was tracked by live-cell imaging for several hours, before DNA replication stalled, and apoptosis ensued (59), akin to uninfected cells (41). Strikingly, short pulses of AO-6MT on VdU-tagged vDNA were well tolerated. Importantly, VdU also incorporated into vDNA of AdV E1-transformed HER-911 or 293 cells, overcoming these cells’ exceptionally narrow tolerance of nucleotide kinases for ethynyl-deoxy-nucleosides. Gratifyingly, the VdU-AO-6MT protocol allowed to click-label AdV vector DNA and produce AdV particles with vDNA that could be directly labeled with AO-6MT without capsid disruption (59). This protocol can now be used to track incoming AdV wild-type and vector DNA at single-molecule sensitivity.

#### Dual-color pulse-chase imaging with CuAAC and IEDDA provides spatio-temporal information on AdV assembly

It has been well established that the assembly of AdV progeny requires intermediate and late viral gene expression, including a primary transcript of the major late transcription unit differentially spliced and polyadenylated yielding L1-L5 mRNAs (120). Intermediate proteins (IVa2, 22K, 33K, pIIIa, 52K where “p” denotes precursor) mediate vDNA packaging and assembly (52K), while late proteins hexon, penton base, pIIIa, fiber, pVI, pVIII, IX, and L3-p23/protease make up the capsid and the DNA-core (IVa2, V, pVII, pX, pTP). Packaged capsids are proteolytically processed by the viral protease L3-p23 into infectious mature virions lacking detectable cellular proteins (121, 122).

The availability of two distinct alkyne-tagged nucleotides for labeling vDNA has allowed for pulse-chase experiments, where an initial EdC pulse was followed by a chase without tagged nucleoside and a later second short pulse with VdU (59) (for illustration, see Fig. 2). It turned out that in this setting, only VdU was found to be incorporated into virion DNA, but not EdC, although both nucleosides were incorporated into replicating vDNA, the first one into early and the second one into late vDNA (59). This reinforces the notion that ongoing viral replication is required for virion formation (123, 124).

**Fig 4:**
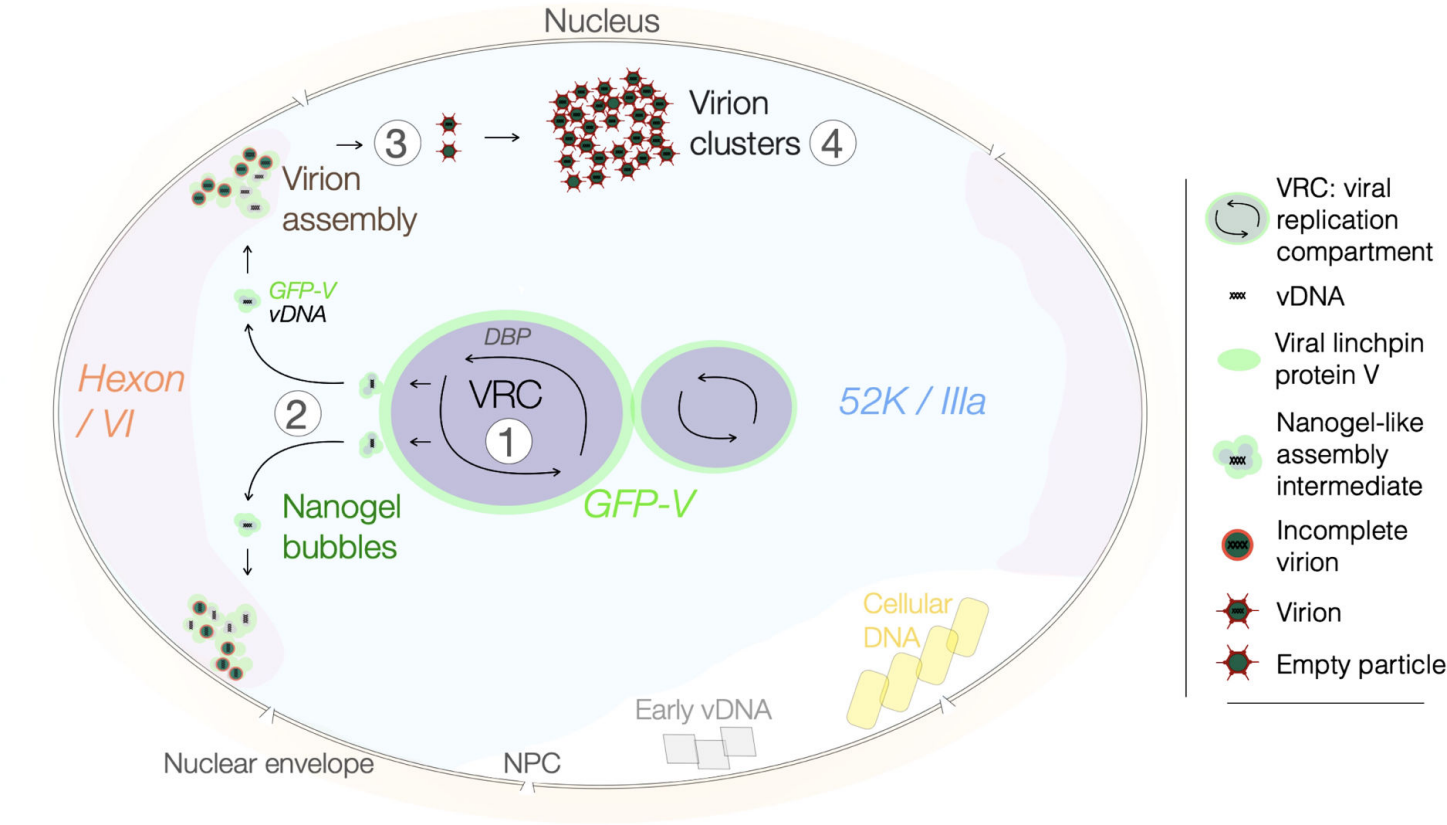
Model derived from click chemistry experiments depicting stepwise assembly of AdV with concurrent vDNA packaging and capsid assembly in the cell nucleus. Dual-label, pulse-chase click chemistry and live-cell confocal, as well as electron microscopy, inform a stepwise concurrent model for AdV assembly in the cell nucleus (see reference 59). The model additionally builds on recent evidence involving the 52K biomolecular condensate in viral assembly (125) and viral assembly intermediates identified by transmission electron microscopy (126). Step 1: the viral replication center constitutes three essential viral proteins, DBP, DNA-Pol, and terminal protein, to amplify vDNA. Step 2: newly replicated vDNA is sequestered from the periphery of the VRC into nanoscale bubbles containing the viral capsid-vDNA linchpin protein V (GFP-V). The bubbles released into the viral 52K domain (containing the viral inner capsid protein IIIa) located around the VRC. Step 3: the GFP-V-vDNA nanogels diffuse in the 52K compartment and cluster in the nuclear periphery, where the major viral capsid protein hexon and its binding partner protein VI are enriched. These loci termed virion formation sites release virus particles that migrate by unrestricted diffusion and eventually form paracrystalline clusters in the nucleus (step 4).

Strikingly, click chemistry and live-cell imaging of the viral linchpin protein V fused to green fluorescent protein (GFP-V), the viral linchpin between vDNA-core and capsid, revealed that newly replicated vDNA bubbled from the VRC surface in a replication-dependent manner (59). A schematic model for AdV assembly derived from click chemistry experiments is depicted in Fig. 4. The bubbled GFP-V nanogels colocalized with CuAAC-accessible vDNA at amorphous structures, as shown by correlative light and electron microscopy. They were embedded in a super-compartment made up of the viral protein 52K containing intrinsicllay disordered regions (IDR), a key factor for packaging and assembly (125). The nanogels exhibited restricted diffusion, suggesting that they represented viral assembly intermediates interacting with components of the 52K compartment (59). They coalesced in the nuclear periphery and released regularly shaped highly diffusive entities 90 nm in diameter. Many of these objects were GFP-V positive and did not stain with CuAAC, suggesting that they were assembled virus particles, while others were both GFP-V and vDNA positive, suggesting incompletely formed particles.

Besides supporting a stepwise assembly model with concurrent vDNA packaging and capsid formation, the dual-color IEDDA and CuAAC protocol also uncovered functionally diverse pools of vDNA in the nucleus. It showed that early-replicated vDNA segregated away from the VRC, became transcriptionally inactive, and was not incorporated into progeny (59). Together with previous observations of protein VII- and histone-packed vDNA coexisting in the same nucleus (109, 127, 128), these data suggest that replication and virion formation are spatially and temporally distinct processes (59, 125, 126). Future studies exploring virion packaging at the interface between VRC and 52K compartment, along with the maturation of the GFP-V-vDNA nanogels, will be necessary to unravel the exquisite selectivity and efficacy of AdV packaging and particle formation.

### ClickSeq probing of RNA virus uncoating *in vitro*

To gain insight into the mechanisms of viral nucleic acid packaging, it can be informative to consider the reaction opposite to packaging, virus uncoating and decondensation. Virions shield their genetic information and can be triggered to release it by a range of cues depending on the virus (for reviews, see references 129, 130). Cues include receptor binding, acidic or alkaline pH, reduction in sodium or calcium ions, limited proteolysis, or temperatures above levels physiological to the agent. Early experiments with UV-psoralen crosslinking and heat treatment of picornavirus RNA showed that the poly-(A) tail at the 3´-end of the single-stranded RNA exits the capsid before the rest of the RNA (131), suggesting polarized vRNA packaging. For an in-depth discussion of picornavirus uncoating, see references 132–135.

To obtain subgenomic resolution of protein interactions with the viral genome, a click chemistry sequencing (ClickSeq) protocol was developed for flock house virus (FHV), a nonenveloped insect virus of the *Nodaviridae* with two distinct genome segments of single-strand positive-sense RNA (Fig. 3D). This ClickSeq protocol has uncovered distinct steps in heat-induced particle disassembly by using randomly primed reverse transcription reactions with azido-2´,3´-dideoxynucleotides that terminate DNA synthesis and release 3´-azido-blocked cDNA fragments (136). Purified fragments are click-ligated via CuAAC to DNA oligonucleotides modified with a 5´-alkyne group, yielding a triazole-linked DNA backbone suitable for PCR amplification and cDNA library generation for RNAseq. The approach is particularly powerful if combined with UV light-induced crosslinking of thio-uridine to nearby protein, and next-generation sequencing and bioinformatics analyses (61). In case of FHV, the RNA release was ordered, that is, the 5´ and 3´ terminal sequences and other loci within the RNA were exposed first, indicating that RNA assumes a rather active role in uncoating (61). The ClickSeq study also revealed an anti-correlation of genome release with RNA-capsid interactions, that is, regions of genomic RNA exhibiting weak interactions with capsid proteins were released first.

Uncoating studies by ClickSeq have the potential to enhance our understanding of the physical properties of virions and inform on virus entry and uncoating processes (102, 137). For example, they could contribute to mapping the interactome of the viral genome within the capsid. ClickSeq could further be correlated to atomic force microscopy (AFM) indentation studies or heat-stress experiments. AFM studies with single AdV particles, for example, showed that enhanced genome condensation in immature virions reduces the brittleness of the capsid and yields softer capsids that cannot respond to the uncoating cues from the cell and, thus, remain noninfectious, unlike wild-type ones (138). Likewise, undersized gutless AdV vectors lacking all viral protein coding sequences and just encoding a GFP reporter gene cassette were less heat stable than nearly full-sized gutless vectors, suggesting that the size of the packaged genome affects thermostability (139). Whether capsid brittleness and temperature resistance are governed by genome interactions with capsid proteins could be tested by ClickSeq. In addition to informing on virion uncoating, ClickSeq could also be applied to the evolving concept of concerted virus particle assembly. In such model, preexisting capsid components and viral genomes get together in a hierarchical manner governed by distinct affinities, for example, high-affinity protein-nucleic interactions forming first, followed by lower-affinity ones.

### Biochemical bulk studies

Viral nucleic acids are variably processed in cells. Besides biochemical modifications, such as methylation or processing by nucleases, they are condensed into viral particles and unpacked from viral particles. Activities of unpacked genomes are regulated by a range of host and viral proteins. For example, proteins associated with viral genomic DNA can be probed by classical chromatin immunoprecipitation studies (for example, reference 140), but this approach is limited by the availability of high-affinity antibodies and by the antibody epitopes being exposed on the proteins. In contrast, iPOND (isolating proteins on nascent DNA) is a highly unbiased approach used to identify viral and cellular proteins associated with viral nucleic acids (141, 142). iPOND uses ethynyl-modified nucleoside analogs to tag nascent DNA for subsequent isolation of the replicated DNA and associated proteins by biotin-azide and CuAAC, followed by quantitative mass spectrometry for relative and absolute quantification (Fig. 3A). It has helped to identify proteins associated with nascent DNA of AdV, HSV-1, vaccinia virus, and polyoma virus (PyV) (143–147). It has allowed for the isolation of HSV-1 replication forks and entire genomes, as well as identification of a cellular restriction factor bound to HSV-1 DNA under conditions that did not express the viral E3-ubiquitin ligase ICP0 (148). In the case of AdV, iPOND was used in combination with systems proteome analyses to compare overall cellular proteome and vDNA-associated proteome in wild-type and early region 4 (E4) protein mutant virus infections (145). E4 encodes viral proteins needed for productive infection, and the study identified potential cellular restriction factors that were degraded in wild-type infection but were found associated with viral genome in the E4 mutant virus infection. In case of vaccinia, iPOND demonstrated the association of the complete set of viral replication proteins with nascent DNA (144), and for HSV-1, AdV, and PyV, it showed that the vDNA-associated proteomes were remodeled in the course of infection (143, 145, 146). With PyV, the protein profile on nascent vDNA at two hours post-synthesis showed that the vDNA contained less DNA primase, DNA-polymerase alpha, or DNA ligase than during synthesis, although functional annotations of these observations remains to be provided (146).

## CLICKING PROTEINS IN VIRAL INFECTIONS

Conformational and subcellular dynamics of proteins is fundamental to cellular and viral functions (for reviews, see, e.g., references 149, 150). Protein mobility emerges as a cornerstone in pathology. A concept called “proteolethargy” entails reduced protein mobility triggered by a range of stimuli, including inflammation (151). Such protein features can be addressed by biosynthetic approaches using click chemistry. Click procedures are increasingly developed to elucidate protein-protein interaction networks in normal cells, immunotherapies or cancer treatments, as well as small molecules interacting with proteins (152, 153). For viral proteins, early experiments directly incorporated the methionine analogue L-homopropargylglycine (HPG) into HSV-1 proteins after host translation shut-down (154). This was followed by CuAAC and provided microscopic insight into protein flux in infection, in particular small nuclear foci of newly synthesized protein domains that progressively increased in size and number during infection. Likewise, HPG has also been used to identify an essential nuclear compartment for the assembly of AdV progeny, formed by the viral intrinsically disordered protein 52K (125).

Besides tagging proteins unselectively, site-specific modifications by genetic code expansion have been developed. This strategy incorporates noncanonical amino acids (ncAAs) into viral proteins by amber stop-codon suppression and cell-specific expression of engineered aminoacyl-tRNA synthetases. For example, it allows for directly labeling proteins with fluorescent dyes compatible with live-cell and super-resolution imaging (155, 156) (Fig. 3E). The procedure entails that an ncAA with a clickable tag is introduced into the sequence of a protein, and then site-specifically modified with a fluorescent dye, for example. For a review on protein labeling strategies and functionalization of viral components, see reference 157.

In the case of bio-orthogonal amino acids (30), the amber-based approach allowed click-labeling of cell surface HIV-1 Env with synthetic dyes and gave insight into nanoscale dynamics by fluorescence recovery after photobleaching and super-resolution microscopy (158). Another example combining genetic code expansion and click chemistry by strain-promoted IEDDA labeled the HIV-1 capsid protein (CA) with a tetrazine-derivative of silicon rhodamine and allowed direct visualization of incoming cone-shaped viral capsids by fluorescence microscopy and correlative imaging (159). Results from this low-invasive study provided evidence for the transport of intact capsids into the nucleus. Given that amber suppression efficiency is generally rather inefficient, and multiple amber stop codons are present in viral and cellular genomes, amber triplets outside of HIV-1 Env were removed from the proviral DNA by mutagenesis (160). This allowed for an increase in the yield of virions and allowed minimally invasive single-molecule Forster resonance energy transfer experiments of Env in HEK293T cells transfected with amber-tagged Env, amber suppressor tRNA, and tRNA synthetase encoding plasmids in the presence of TCO-tagged lysine (160). Env dynamics were analyzed on the surface of infected cells or HIV particles upon conjugation with suitable tetrazine-dyes, showing that Env is a collective of dynamic conformational states conducive for functions in virus entry and egress. Clearly, the combination of click chemistry with genetic code expansion can overcome background fluorescence problems in imaging and offers an essentially tag-free approach to studying biophysical organization of proteins in time and space. Yet, it remains to be seen how this elegant labeling approach will be adopted by the broad virology community, given that it requires virus-optimized ncAAs and tRNA/aminoacyl-tRNA synthetase pairs.

## CLICKING LIPIDS

Lipids are a key building block of membranes, where phospholipids, sphingolipids, and cholesterol together maintain the integrity of cellular membranes and enveloped viruses (161–163). Lipids and their metabolites control homeostasis and disease. For example, high levels of the sphingolipid ceramide (Cer) induce apoptosis (164), and uncontrolled levels of Cer correlate with reduced activity of sterol regulatory element-binding protein (SREBP) and decreased cholesterol *de novo* synthesis (165). This situation is reminiscent of certain forms of lysosomal storage disease, for example, caused by nonfunctional Niemann Pick type C1/2 proteins, where both SREBP and acetyl-CoA-acetyltransferase (ACAT) activities are dysregulated (166, 167). Restoring ACAT activity under conditions of deregulated cholesterol homeostasis is one of the functions of the AdV E3 protein RIDa (receptor internalization and downregulation alpha) (168).

Unlike proteins and nucleic acids, lipids behave more like metabolites or small-molecule drugs than nucleic acids and proteins do. This, together with the small size of lipid molecules, has made it difficult to label lipids without disturbing their trafficking and metabolism. In addition, while relatively few lipids work as single entities, for example, as small covalent additions in lipidated proteins (169, 170), the vast majority functions as an ensemble through low-affinity interactions with other lipids or proteins in membrane bilayers or monolayers on lipid droplets (171, 172). Furthermore, the chemical space of lipids is immense, as indicated in the LIPID MAPS database comprising tens of thousands of lipids (www.lipidmaps.org) (173). Certain lipids, such as phospholipids or sphingolipids, have proviral as well as anti-viral functions by building platforms for virus replication or cell entry, or they enhance anti-viral innate immunity (174–176).

Lipid flux and subcellular localization are increasingly addressed with clickable lipid probes, also in combination with mass spectrometry and multiplexing gaining high sensitivity (177–179). Besides clickable tags, other chemical modifications boost the delivery of lipid probes into cells or protect the probe from rapid metabolization. For example, esterified polar head groups or charged residues can be readily uncaged by cytosolic esterases or UV-light treatment removing, for example, protective coumarin or nitrobenzyl groups (180). Importantly, photo-crosslinking combined with fluorophore or biotin clicking strongly enhances the accuracy of subcellular analyses, or lipid-binding protein pulldown from cell lysates upon trypsinization and tandem mass spectrometry (180, 181).

### Clickable lipid probes in HSV-1 infection

Clickable lipid probes are providing mechanistic insights into lipids in virus infections (180, 182, 183). For example, minimally modified alkyne- or azido-tagged aliphatic chains are suitable for metabolic flux studies, as they are well-established substrates for lipid processing enzymes, such as sphingomyelinase, which cleaves sphingomyelin (SM) to phosphatidylcholine and Cer (reviewed in reference 181). Upon cell fixation, such lipid probes can also be coupled to fluorophores or affinity labels for fluorescence tracking and localization, or proteomic and lipidomic analyses of interaction partners. The former reaction occurs through CuAAC and the latter by SPAAC, which is widely used in lipid research (40) (Fig. 3F and G).

The power of clickable lipids was exemplified by the finding that immortalized human keratinocytes rapidly turned alkyne-tagged sphingosine (Sph) into Cer and then SM, but that HSV-1 infection inhibited the conversion of Cer to SM (184). Sphingolipid profiling showed that the tagged Cer species was used by ceramide transfer protein (CERT) to synthesize SM. CERT mediates nonvesicular Cer transport from the endoplasmic reticulum to the trans-Golgi network, where Cer is metabolized to SM and other sphingolipids with important functions in proliferation, signaling, and membrane trafficking (185). CERT was found to be activated by pUL21 of HSV-1 (184), a protein phosphatase adaptor boosting the dephosphorylation of cellular and viral proteins, including CERT. pUL21-mutated HSV-1 lacking CERT activation was even more compounded in SM synthesis from Cer than wild type HSV-1. This suggests that Cer to SM conversion is beneficial to HSV-1 infection, although virus replication and spread were found to be unaffected by pUL21-mediated CERT dephosphorylation.

Another example illustrating the power of clickable lipid probes is azido-tagged Sph. Sph is a breakdown product of Cer by endo-lysosomal acid ceramidase (aCDase) and accumulates in lumenal vesicles of endosomes. aCDase is a prominent genetic determinant for Farber disease, a lysosomal storage disorder in humans (186). Studies of HSV-1 entry into macrophages showed that Sph functions as a trap for endocytosed HSV-1 (187). In the presence of aCDase and Sph, viruses fuse with Sph-rich lumenal vesicles instead of the limiting endosomal membrane and are targeted to degradation in lysosomes. These data support the notion that lipids and their metabolites are becoming increasingly important multi-functional drug targets, as indicated by newly synthesized fatty acids from fatty acid synthase, a driver of virus infections, cancer, and metabolic dysfunction-associated steatohepatitis (188–190). In sum, lipid-centric views of infection are underexplored. Click chemistry in lipid research promises to give new insight into virus-controlled organelle dynamics and infection mechanisms.

## CONCLUSION AND PERSPECTIVES

Click chemistry has had a major impact on molecular cell biology as well as fundamental and applied virology. While initial applications in virology have covalently labeled DNA and RNA in the test tube (191), an increasing range of functionalized reporter molecules (ligands, labels, fluorophores) has been developed to click-label nucleic acids, proteins, and lipids in both fixed and live cells. This has informed new models for virus entry, uncoating, replication, and assembly (Fig. 4). Besides ultrasensitive tracking of nucleic acids, proteins, and lipids as discussed in this review, click chemistry has also catalyzed the development of new anti-viral approaches, for example, chemicals covalently modifying HIV capsid and blocking viral assembly (192), or high-sensitivity quantification of HIV-1 p24 antigen in clinical samples (193).

There is no doubt that click chemistry applications will continue to be immensely useful for a better understanding of all phases of the viral replication cycle, including nucleic acid packaging and particle assembly processes. This lays a foundation for exploring infection variability and viral disease (27, 194). In addition, click chemistry will be instrumental in refining information on the topological organization of packaged viral genomes, including protein contact sites to nucleic acids, virion stability, and the energetics of condensation and decondensation in assembly and uncoating. Click chemistry will continue to provide fresh opportunities to conceive new preventive and curative treatments against viruses, also in conjunction with physical properties of virions and intermediate structures reflecting stepwise changes of virions during cell entry, assembly, and maturation processes (102, 137, 195). This may go along with a deeper exploration of the role of lipids in organizing VRCs and promoting viral transmission (180, 196). We are looking forward to continuing collaborations between virologists and chemists that may provide novel insights into the nature of infectious diseases.
